# Supplementary material for: Ibuprofen, Flurbiprofen, Etoricoxib or Paracetamol Do Not Influence ACE2 Expression and Activity In Vitro or in Mice and Do Not Exacerbate In-Vitro SARS-CoV-2 Infection
Source: Int J Mol Sci. 2022 Jan 19;23(3):1049. doi: 10.3390/ijms23031049 (PMC8835123; doi:10.3390/ijms23031049)
Supplement: Supplementary file 1 [file ijms-23-01049-s001.zip › ijms-1537498-supplementary-update.pdf]

International Journal of
Molecular Sciences

MDPI

Article

Ibuprofen, Flurbiprofen, Etoricoxib or Paracetamol Do Not Influence ACE2 Expression and Activity In Vitro or in Mice and Do Not Exacerbate In-Vitro SARS-CoV-2 Infection

Table S1. Statistical results ACE2 mRNA (A) or protein (B) levels of heart, lung and aorta and (C) Angiotensin 1-7 plasma levels. Each treatment group consists of 8 mice.

	**ACE2 mRNA, quantitative PCR (A)**		
	**Heart**	**Lung**	**Aorta**
**Outliers**	No	No	1 outlier (IBU 50)
**Norm. distr.**	Yes	No	No
**Test**	ANOVA	Kruskal-Wallis H	Kruskal-Wallis H
**F/H**	$F(3,31) = 0.297$	$H(3) = 4.112$	$H(3) = 2.950$
**p**	$p = 0.827$	$p = 0.250$	$p = 0.399$
**Sign. Different**	No	No	No
	**Protein levels, western blot (B)**		
	**Heart**	**Lung**	**Aorta**
**Outliers**	No	No	1 outlier (IBU 50)
**Norm. distr.**	Yes	Yes	No
**Test**	ANOVA	ANOVA	Kruskal-Wallis H
**F/H**	$F(3,31) = 1.636$	$F(3,31) = 1.507$	$H(3) = 1.246$
**p**	$p = 0.204$	$p = 0.234$	$p = 0.742$
**Sign. Different**	No	No	No
	**Plasma Angiotensin 1-7, ELISA (C)**		
**Outliers**	No		
**Norm. distr.**	Yes		
**Test**	ANOVA		
**F/H**	$F(3,31) = 0.644$		
**p**	$p = 0.593$		
**Sign. Different**	No		

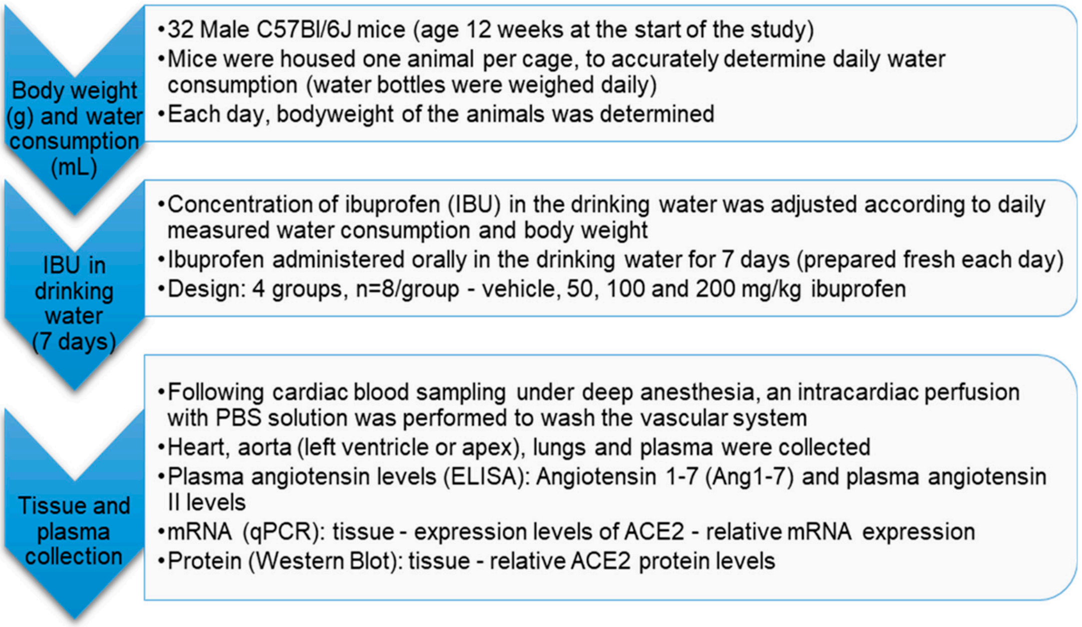

Figure S1. Flow chart of ibuprofen pharmacokinetic study.
